# HEPATECTOMY FOR PYOGENIC LIVER ABSCESS TREATMENT: EXCEPTION APPROACH?

**DOI:** 10.1590/0102-672020180001e1394

**Published:** 2018-08-16

**Authors:** Sergio Renato PAIS-COSTA, Sergio Luiz Melo ARAUJO, Victor Netto FIGUEIREDO

**Affiliations:** 1 Hospital Santa Lucia, Brasília, DF, Brazil

**Keywords:** Laparoscopy, Hepatectomy, Liver abscess/surgery, Laparoscopia, Hepatectomia, Abscesso hepático/cirurgia

## Abstract

**Background:**

Percutaneous drainage for pyogenic liver abscess has been considered the gold-standard approach for the treatment on almost of the cases. However, when percutaneous drainage fails or even in some especial situations, as multiloculate abscess, lobe or segment surgical resection can solve infectious clinical condition.

**Aim:**

To report a series of patients who underwent hepatectomy for pyogenic liver abscess performed by a single surgical team.

**Methods:**

Eleven patients were operated with ages ranging from 45-73 years (mean and median 66 years). There were eight men and three women. The etiologies were: idiopathic (n=4), biliary (n=2), radiofrequency (n=2), direct extension (n=1), portal (n=1), and arterial (n=1). The mean lesion diameter was 9.27 cm (6-20 cm).

**Results:**

The mean operation length was 180 min (120-300). The mean intra-operative blood loss was 448 ml (50-1500). Surgical approaches were: right hepatectomy (n=4), left hepatectomy (n=3), left lateral sectioniectomy (n=1), right posterior sectioniectomy (n=2), resection of S8 (n=1), and S1 (n=1). Postoperative morbidity rate was 30%, while mortality was null. Median hospital stay was 18 days (5-45). The median follow-up period was 49 months (13-78). There was single lesion recurrence.

**Conclusion:**

Hepatectomy can be done as exception approach for pyogenic hepatic abscess treatment; it is a good therapeutic option in special situations.

## RESUMO

**Racional:**

A drenagem percutânea do abscesso hepático piogênico tem sido considerada padrão-ouro para o tratamento na maioria dos casos. Mais raramente, quando essa abordagem falha ou em casos de abscessos multiloculados, a ressecção daquele segmento ou lobo destruído pode resolver o contexto infeccioso.

**Objetivo:**

Relatar uma série de doentes com abscesso hepático que foram submetidos a hepatectomia.

**Método:**

Onze doentes foram operados. A idade variou de 45-73 anos (média e mediana 66). Foram oito homens e três mulheres. As causas foram: idiopática (n=4), biliar (n=2), radiofrequência (n=2), portal (n=1), arterial (n=1), e extensão direta (n=1). A média do tamanho das lesões foi 9,27 cm (6-20).

**Resultados:**

A média de tempo cirúrgico foi de 180 min (120-300). A média de sangramento intra-operatório foi de 448 ml (50-1500 ml). Os procedimentos cirúrgicos foram: hepatectomia D (n=4), hepatectomia E (n=3), setorectomia lateral E (n=1), setorectomia posterior D (n=2), ressecção do S8 (n=1) e S1 (n=1). A morbidade pós-operatória foi 30%, enquanto a mortalidade foi nula. Mediana de internação foi de 18 dias (5-45). A mediana de seguimento foi 49 meses (13-78). Houve uma única recidiva de lesão.

**Conclusão:**

Embora a hepatectomia possa ser considerada conduta de exceção para tratamento do abscesso hepático piogênico, ela deve ser boa opção terapêutica em situações especiais.

## INTRODUCTION

Hippocrates first described pyogenic liver abscess (PLA) around 400 BC, but Oschneret al.^13^ reported one series of 47 cases associated with appendicitis. PLA is a rare condition with significant geographic variation, with a reported annual incidence of 3.6 cases per 100.000 individuals in the United States, but up to 17.6 per 100.000 in Taiwan. There is a slight predominance in males. Due to changes in etiology, PLA now primarily affects older individuals, with peak incidence between 50-60 years^8^.

Nevertheless, in the past the main cause of PLA was pylephlebitis due acute appendicitis; nowadays, the causes have been varied worldwide. Biliary tract disease has been reported as the most frequent source followed by septic embolus by portal or arterial circulations, cryptogenic, trauma or more unfrequently after ablation by radiofrequency of hepatic tumors^5^ ^-^ ^12^.

Symptoms are diverse, as fever, abdominal pain, chills, jaundice, ascites, or pleural effusion^9^. If untreated, PLA invariably is lethal. Currently, even with substantial increase of therapeutic approach, overall mortality may vary around world. In more recent series, mortality rates of 1-31% have been reported globally^18^.

Management of this disease varies considerably from surgeon to surgeon. Nonetheless, in the past open surgery approach was preferential; nowadays, percutaneous drainage has becoming the main therapeutic approach. It is very effective and presents low morbidity, nonetheless sometimes fail or even difficult to be performed. On these special circumstances, surgical drainage should be indicated. In other hand, when PLAs are multiple, multiloculate or present solid areas with destruction of adjacent hepatic parenchyma up front surgery for drainage or even hepatic resection has been advisable^1^ ^-^ ^3^ ^,^ ^5^ ^-^ ^12^ ^,^ ^16^ ^-^ ^19^.

The aim of this study was to describe a series of PLA that underwent formal hepatic resection of committed segment(s) or even entire lobe as therapeutic approach. 

## METHODS

Between January 2008 and July 2015, 11 hepatectomies for PLA treatment were carried out at Santa Lucia Hospital, Brasilia, DC, Brazil. All of the resections were performed by a single surgical team. Clinical presentation, past medical history, and microbiological and radiological parameters were obtained from each patient’s record. This data included age, gender, type of hepatectomy, symptoms, co-morbidities, ASA score, underlying etiology, presence of diabetes mellitus, BMI, presence of malignancy, imaging studies focused on the size and number of abscesses, microbiological findings, and the abscess components (fluid, gas, walls). The size of the abscess was defined as the largest diameter reported on CT. The bacteria responsible for the PLA were also noted. The biological parameters examined included hemoglobin and albumin levels, white blood cell count, creatinine level, CRP, fibrinogen, total bilirubin level, and liver enzymes. When malignant disease was suspected assays for the tumor markers CEA, AFP and Ca 19.9 were done.

The etiology of the abscesses was classified into four groups: biliary, portal, cryptogenic, and others (after hepatic procedures, arterial, and direct extension for example). The choice of initial treatment was based on the preference of the attending physician. The treatments were antibiotics for six weeks and percutaneous treatment in cases which was possible. Surgical treatment was preferentially chosen on these circumstances: lack of percutaneous drainage or when it failed, cases of muliloculate abscesss or solid-areas, multiple small abscesses which were confined in one lobe without response to initial antibiotics, and concomitant surgical diseases. Surgical treatment was performed by abscess unroofing or even formal hepatic resection. When unroofing failed, the procedure was transformed in hepatectomy (after percutaneous drainage or not). Failure of antibiotic or percutaneous treatment was defined as persistent infection after 3-5 days of the chosen therapy. Treating the cause of the abscess was also recorded. Cure was defined as resolution of the infectious syndrome and symptoms at two months after initiating treatment. Septic shock was defined as a state of combined hypotension, tachycardia, polypnea, oliguria, and altered mental status with a temperature over 38^◦^ C or under 36^◦^ C. Mortality was defined as death within 30 days or during the concurrent hospital admission. The first choice of surgical access used to be the one for hepatectomy, considering laparoscopic approach whenever feasible. 

Always, for the laparoscopic approach were performed standard techniques reported previously by the present authors^4^ ^,^ ^14^ ^-^ ^15^. All patients had histological examination of the lesion to exclude malignant tumor and to evaluate presence of *Entomoeba hystolitica*.

## RESULTS

Majority of patients were male (72%) and elderly people with mean age of 66 years (45-73). Main associated diseases were the following: diabetes (45%), hypertension (36%), obesity (18%), and colorectal cancer (18%). Clinically were observed: fever (100%), right upper superior quadrant pain (90%), chills (82%), and palpable mass (63%). Three patients presented sepsis (27%) on their initial attendance at hospital. There was no case of septic shock. Almost all presented leukocytosis (91%) and hypoalbuminemia (72%). All patients were anemic. Renal failure was observed in 40% of these patients.

**FIGURE 1:**
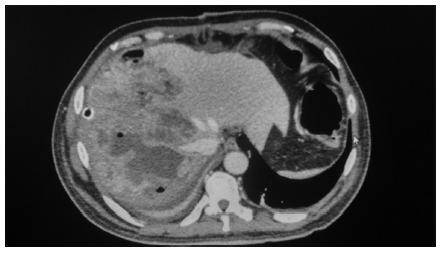
Abdominal CT showing large solid-cystic multiloculate septate liver abscess into right lobe in diabetic patient with sepsis

All patients were submitted to image evaluation. The multislice abdominal CT showed septate solid-cystic multiloculate single liver lesion in five cases (Figure 1), single cystic lesion in three cases, multiple cystic lesions in three cases. Gas- formation lesion was observed in three cases (Figure 1). The right lobe was comprised in six patients (54.5%) while left lobe in five (45,5%). One patient presented bilateral abscesses. Major diameter lesion ranged between 6 and 20 cm (mean 9.27 and median 9.1). 

The etiologies were: idiopathic (n=4), biliary due ascendant cholangitis (n=2), complication after radiofrequency coagulation in colorectal metastasis (n=2), direct extension from acute cholecystitis (n=1), portal due to diverticulitis (n=1), and arterial due endocarditis (n=1). Causative PLA agent was possible be identified in 90% of cases. Isolated bacteria were: *Escherichia coli* (n=5), *Klebsiella pneumoniae* (n=4), and *Bacteroides fragilis* (n=2, Tables 1 and 2)..

**TABLE 1:** Patient characteristics

Case	Gender	Age	Etiology	Number	Diameter (cm)	Localization	Associated disease	ASA	Isolated bacteria
1	F	45	Acute cholecystitis	1	8.5	Left lobe	None	I	E.coli
2	F	70	Cholangitis	4	8.3	Both lobes	Diabetes	II	E.coli
3	M	62	Portal diverticulitis	1	10	Left lobe	Hypertension	II	B. fragilis
4	M	73	Idiopathic	1	7.2	Left lobe	Hypertension + diabetes	III	K. pneumoniae
5	F	54	Idiopathic	2	12.3	Right lobe	Diabetes	II	K. pneumoniae
6	M	61	CRC Mets - RFC	3	12	Right lobe	Diabetes	II	B. fragilis
7	M	66	Idiopathic	1	9.1	Left lobe	None	I	E. coli
8	M	67	CRC-Met-RFC	1	6	Left lobe	Obesity	I	E. coli
9	M	70	Biliary - cholangitis	3	8	Right lobe	Hypertension	II	E. coli
10	M	64	Arterial - endocarditis	1	10.7	Right lobe	Obesity +hypertension	III	-
11	M	66	idiopathic	1	20	Right lobe	Diabetes	II	K.pneumoniae
Median		66	-	1	9.1	-	-	-	-
Mean		66	-	-	9.27	-	-	-	-

ASA= American Society of Anesthesiologists; CRC=colorectal cancer; Met=metastasis; RFC=radiofrequency; E=Escherichia; B=Bacteriodes e K=Klebsiella

**TABLE 2:** Laboratorial findings

Case	Albumin	Hemoglobin	Creatinine	Alkaline phosphatase	Leukogram	Total bilirubin
1	3.3	10.6	1.2	50	23329	1.9
2	3.8	9.7	0.7	213	18575	0.8
3	3.0	8.3	0.5	89	32115	2.6
4	2.6	12.1	1.2	62	15000	1.9
5	3.7	9.8	1.1	53	23450	0.4
6	1.8	10.5	2.9	590	17435	2.8
7	2.8	10.3	1.2	345	21978	1.2
8	3.2	9.7	2.3	900	24590	3.8
9	2.7	8.7	3.5	121	14659	1.2
10	3.1	9.9	4.7	206	22890	1.1
11	3.0	10.9	2.1	167	17800	0.7
Mean	3.0	10.04	1.97	248.72	21074	1.67
Median	3.0	9.9	1.2	167	18575	1.2

**FIGURE 2:**
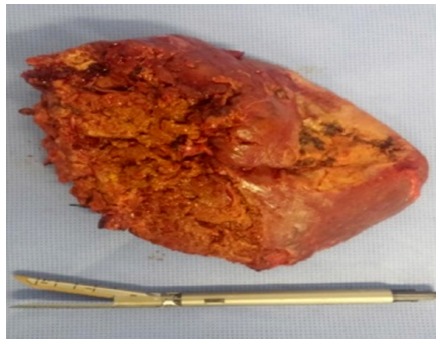
Right lobe with multiseptated liver abscess

**FIGURE 3:**
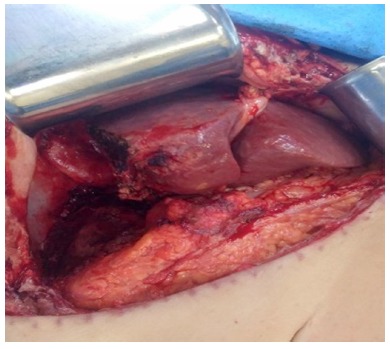
Final aspect of residual left liver after straightforward right hepatectomy by means open approach due multiseptated giant PLA

Surgical intervention (hepatic resection) was just indicated due following aspects: failure of percutaneous drainage (36%), septate solid-cystic multiloculate liver lesions (27%, Figures 1 and 2), multiple cystic lesions without response large spectrum antibiotic therapy (19%), and failure of laparoscopic unroofing for PLA (18%). Open hepatic resection was performed in seven patients (Figure 3). Open procedures were: right hepatectomy (n=4), left hepatectomy (n=1), posterior right sectioniectomy (n=1), and caudate resection (n=1). Laparoscopic procedure was completed in other four patients where none of them underwent open conversion. Laparoscopic resections were: left hepatectomy (n=2), posterior right sectioniectomy (n=1) and left lateral segmentectomy (n=1). One patient with bilateral abscess underwent on same surgical time two laparoscopic surgeries which were following laparoscopic posterior right sectioniectomy and unroofing procedure for two small abscesses in left lobe. As all laparoscopic patients, as three open approach patients were resected without vascular clamping (Pringle maneuver). Three patients submitted to open right hepatectomy underwent hemi-Pringle maneuver and one case (right hepatectomy in obese patient with three previous laparotomies) underwent total vascular exclusion of the liver, in order to control severe intraoperative bleeding of vena cava. Estimated intra-operative blood loss ranged from 50-1500 ml with mean 448,63 and median 325 ml. Surgical time ranged between 120-300 min (median 161). Weight of resected specimen ranged between 150-950 g (mean 397).

Four patients received transfusions in this series; all of them underwent open right hepatectomy (Figure 3). There were three major complications (30%) in three patients, all of them submitted to open approach. There was a single reoperation (9%). Postoperative complications were respectively: one case of evisceration solved by re-suture (Case 6), one of massive intraoperative hemorrhage due vena cava lesion that was controlled by suture and transfusions (Case 10), and finally one case of postoperative infected biloma solved by percutaneous drainage (Case 11).

There was no mortality on this series. All of patients underwent surgical drainage of the liver bed by means of tubular drain, taken out when presented both no-biliary aspect and its debit was lower than 50 ml/24 h for two consecutive days. All patients underwent large spectrum antibiotic therapy for unless 21 days. Multislice CT was done after seven to ten days in postoperative period. In all patients that underwent laparoscopic hepatectomy, the oral intake begun on the 1^st^ postoperative day. Hospital stay ranged between 5-45 days (mean 18). There was infection resolution in all cases. All patients, which were symptomatic, achieved complete symptom relief. The details of the both surgical procedures and short-time outcomes are shown in Table 3.

**TABLE 3:** Surgical characteristics

Case	VC min	Intraoperative blood loss (ml)	Blood transfusions	Operation length (min)	Weight of the surgical specimen (g)	Type of hepatic resection	Postoperative complication	Hospital stay (days)
1	-	100	no	153	353	LLH	-	07
2	-	50	no	161	300	LRPS+unroofing	-	10
3	-	50	no	120	431	LLLS	-	05
4	-	205	no	155	320	LLH	-	05
5	-	720	yes	211	675	ORH	-	25
6	20	330	no	135	256	ORPS	evisceration	23
7	-	150	no	160	397	OLH	-	14
8	30	325	no	180	150	OCL	-	18
9	-	770	yes	185	880	ORH	-	25
10	45	1500	yes	300	950	ORH	hemorrhage	21
11	35	735	yes	225	780	ORH	Infected Biloma	45
Mean	32.5	448.63	-	180.45	497.27	-	-	19.81
Median	-	325	-	161	397	-	-	18

VC=vascular clamping; LLH=laparoscopic left hepatectomy; LRPS=laparoscopic right posterior sectioniectomy; LLLS=laparoscopic left lateral segmentectomy; OLH=open left hepatectomy; ORH=open right hepatectomy; ORPS=open right posterior sectioniectomy; OCL=open caudate lobectomy

Histological examination showed residual adenocarcinoma in both patients that were previously submitted to radiofrequency coagulation of metastasis. Mean and median of follow-up period was 49 months (13-78). There was single abscess recurrence in a difficult control diabetic patient which underwent right hepatectomy (Case 11) after eight months of surgical resection (right hepatectomy). This new abscess was successfully solved by means percutaneous drainage.

## DISCUSSION

PLA is an uncommon disease that generally affects middle age or even elderly people with associated multiple co-morbidities mainly diabetes and hypertension^6^ ^,^ ^7^ ^,^ ^12^. These findings were also observed on this sample, when majority of patients were elderly and unless one third presented diabetes and hypertension. Like reported by Quet al.^17^, PLA was also associated with cancer patients as observed on present casuistic. Clinical findings of PLA may be diverse, nonetheless as pain in right upper abdominal quadrant as fever were frequently found like in other published series^6^ ^,^ ^9^ ^,^ ^16^. As described by different authors, sepsis was a common finding on this sample, present in 30%. Although Alkofer et al.^1^ had observed 10% with septic shock at the hospital admission, on this series none was observed. 

As well as reported by other authors, on this casuistic was observed that almost all patients presented both leukocytosis (91%) and hypoalbuminemia (72%)^1^ ^,^ ^12^ ^,^ ^16^. Less commonly was also observed renal insufficiency with high levels of serum creatinine as observed by Onder et al.^12^. Other laboratorial alterations, as abnormal elevated levels of bilirubin, alkaline phosphatase or even tumor markers (CEA or CA 19,9) has been also found by different authors ^7-9^

Besides both clinical and laboratorial diagnostic from PLA, the cornerstone to identify this affection has been radiologic examinations. Despite abdominal ultrasonography can be important in the screening, the main diagnostic method has been abdominal CT. Generally, a cystic hepatic focal lesion with occurrence of capsule contrast impregnation or non-enhancing hypodense lesion with rim enhancement on CT, is observed in PLA cases. However, more rarely, solid-cystic lesion with debris or gas may be found^2^ ^,^ ^6^ ^,^ ^8^. Like observed by others, the right lobe was more frequently affected by PLA than left^1^ ^,^ ^2^ ^,^ ^9^ ^,^ ^12^. As well as Onder et al.^12^, it was observed about 10% of bilateral PLA.

Different etiologic causes have changed over last years, since when it was described by Oschner et al.^13^ in 1938; at that time the main source was pylephlebitis due appendicitis or more rarely diverticulitis by portal via. However, since the evolution of both advances of surgical practices and best knowledge of the microbiology associated yet ameliorate of large spectrum antibiotic therapy over time, its frequency as the primary source of PLA has been decreased^6^. Subsequently, pylephlebitis has been replaced by ascendant biliary infection as cholangitis (due either choledocolithiasis or malignant neoplasms) or even direct extension due acute cholecystitis. Nowadays, although the causes might be diverse, it has been observed increasing of cryptogenic etiology that has ranged between 18-66% of total cases of PLA around the world^8^. At present series like was also observed by Mangukya et al.^9^, the main cause of PLA was cryptogenic followed biliary causes.

Interestingly, there were two colorectal cancers (20%) when the etiology was related as a late complication after ablation by radiofrequency for treating its metastasis. Both patients, who underwent radiofrequency coagulation ablation of colorectal metastasis, were concurrently submitted to formal hepatectomy by open via to treat bilateral metastasis. Both patients underwent multiple cycles of chemotherapy associated biologics agents. The first one was a malnourished diabetic patient which was submitted to formal open left lateral segmentectomy with radiofrequency coagulation ablation of three lesions in segments VI-VII and second one superobese patient which underwent right hepatectomy with radiofrequency coagulation of one lesion in caudate lobe. Both patients presented important post-chemotherapy hepatic steatosis due irinotecan abusive use. Association of PLA and cancer, mainly colorectal has been also reported over last years^17^. In author’s viewpoint, perhaps these conditions might have contributed to PLA establishment. Since radiofrequency coagulation was initiated for treating hepatic tumors, PLA has been described as possible postoperative complication of this therapeutic approach on the literature^10^. More recently, Pang et al. had reported incidence about 10% of PLA in association with its use. Direct extension of inflammatory process, as cholecystitis, has been also associated with PLA, mainly in diabetic patient with gallbladder empyema like observed on this sample. Even though, pylephlebitis by diverticulitis might be common cause, and it was observed in a single case on the present casuistic. Hepatic arterial seeding has been described as cause of PLA, mainly in patients which underwent use of immunosuppressant and arterial chemoembolization. However, PLA might be associated with endocarditis as observed on this sample; this etiology has been considered a relatively rare phenomenon^18^.

The microbiology varies by etiology and geography. Most PLA are multimicrobial, with commonly identified pathogens including mixed enteric facultative and anaerobic species. In western series, the most commonly isolated organism is *Escherichia coli*, followed by *Kebsiella pneumoniae*, *Enterococcus* and *Streptococcus* species^8^. The present findings were similar to literature^7^ ^,^ ^9^ ^,^ ^16^ ^,^ ^18^.

Despite surgical treatment for PLA be very effective, and much used in the past, actually this concept has significantly changed over time^1^. Nowadays, PLA management has been mainly done by means both percutaneous drainage and intravenous antibiotics with high levels of safety and efficacy^7^ ^,^ ^9^. Even though a non-operative interventional radiology approach has become the first therapeutic choice for PLA, surgical treatment is still necessary in some cases. About 7-58% of PLAs have required surgical treatment^1^ ^,^ ^6^ ^,^ ^7^ ^,^ ^9^ ^,^ ^16^. The main causes of surgical management have been: rupture of PLA with peritonitis, inappropriate local or failure of percutaneous drainage, multiloculate or septate abscesses, multiple abscesses, and PLA with solid content^1^ ^-^ ^3^ ^,^ ^5^ ^-^ ^9^ ^,^ ^12^ ^,^ ^16^ ^-^ ^19^.

The main cause of surgical approach at present series was failure of percutaneous drainage, like observed by Alkof et al.^1^ where one third of operated patients had this situation. Those authors found factors associated to failure in non-surgical treatment, in order to gas-forming PLA and severe sepsis. The second cause of surgical indication found by present authors was solid-multiloculate lesion with a subjacent destroyed liver parenchyma due PLA in 27% of cases followed of multiple lesions without response to antibiotic therapy (19%) and failure of surgical PLA unroofing (18%). Multiloculate lesions are very difficult for doing an efficient drainage by means of percutaneous approach; so, in our viewpoint, early surgical resection on affected segment or lobe solves very quickly infectious complications, as observed on this series. Alkofer et al^1^ observed high solubility when early surgical intervention was performed at similar situations, where 21% needed a formal hepatectomy for solving PLA and sepsis. 

Overall morbidity of present series was high, about 30%; nonetheless, when we consider only patients which were submitted to an open approach, morbidity was similar to Onder et al. ^12^. Onder et al.^12^ observed 42% of morbidity only in open approach, without referring surgical procedure used (hepatic resection or unroofing). At present series, all of cases which presented complications were just those operated by means of open approach. Nevertheless, when was considered only laparoscopic approach the morbidity decreased to 0%. This finding seems favor laparoscopic approach due lower morbidity. Causes of morbidity are associated to clinical conditions and extensive surgery. At present series, the cases which underwent complications were respectively: one case of evisceration in diabetic and malnourished colorectal cancer patient that underwent to open right sectioniectomy, also requiring reoperation (Case 6), and one of massive intraoperative hemorrhage due vena cava lesion that was controlled by suture and transfusions in an obese patient with strong adherences related to three previous laparotomies for treating complicated colorectal cancer (Case 10). One case of postoperative infected bilioma was solved by percutaneous drainage and endoscopic papillotomy (Case 11).

Nonetheless, the present morbidity was high, but overall mortality was null like recently observed by Heneghan et al.^6^ and Tu et al. ^19^. On comparative analysis between surgical (21% of hepatectomies on this sample) and percutaneous or only antibiotics groups, Alkofer et al.^1^ have showed 2.3% (Surgical Group) against 10% (Others No-Surgical Groups) of overall mortality. However, in our viewpoint, perhaps a selection bias could be present because worst patients were selected to non-surgical approach groups. After multivariate analysis, these authors have concluded by means ROC curve application that two variables were associated with mortality, hemoglobin and albumin levels. The cut-off for death was in order to 9.5 gr-dl for hemoglobin and 2.1 gr-dl for albumin. In this series, overall mortality was low because patients presented reasonable clinical conditions without shock with relatively high levels of both hemoglobin and albumin, besides they had been managed by means early surgical approach. 

## CONCLUSION

Nevertheless hepatic resection is an exception approach for pyogenic hepatic abscess, it is a good therapeutic option that should be considered in special situations.
